# Supplementary material for: Aging-related peroxisomal dysregulation disrupts intestinal stem cell differentiation through alterations of very long-chain fatty acid oxidation
Source: PLoS Biol. 2025 Dec 19;23(12):e3003552. doi: 10.1371/journal.pbio.3003552 (PMC12716710; doi:10.1371/journal.pbio.3003552)
Supplement: S1 Fig — (A) Treatment of Drosophila with 50 or 500 µM aspirin failed to restrain the age-associated ISC differentiation defect. (B) Excretion of Drosophila treated with Bromophenol blue. (C) Representative images and quantification of Drosophila midguts treated with the pH indicator Bromophenol blue. There are three conditions: Homeostasis, a well-defined acidic (yellow colored) in copper cell region (CCR), and anterior midgut (AM) and posterior midgut (PM) is basic (blue colored); “Perturbed” the acidic region is lost and the whole gut is basic; “No eating”, no Bromophenol blue showed in the midguts. (D) Representative images of “Smurf” flies after consuming a non-absorbed food dye. (E) Aspirin treatment extended the life span of male Drosophila. (F, G) Varying Aspirin concentrations (2 µM/20 µM/200 µM/2 mM/20 mM) on mouse intestinal organoids culturing. Error bars represent SDs. Student’s t tests, one-way ANOVA, Kruskal–Wallis test, and log-rank test, *p < 0.05, **p < 0.01, ***p < 0.001, ****p < 0.0001, and NS (non-significant) represents p > 0.05. Underlying data and statistical analysis in S8 Data. (DOCX) [file pbio.3003552.s001.docx]

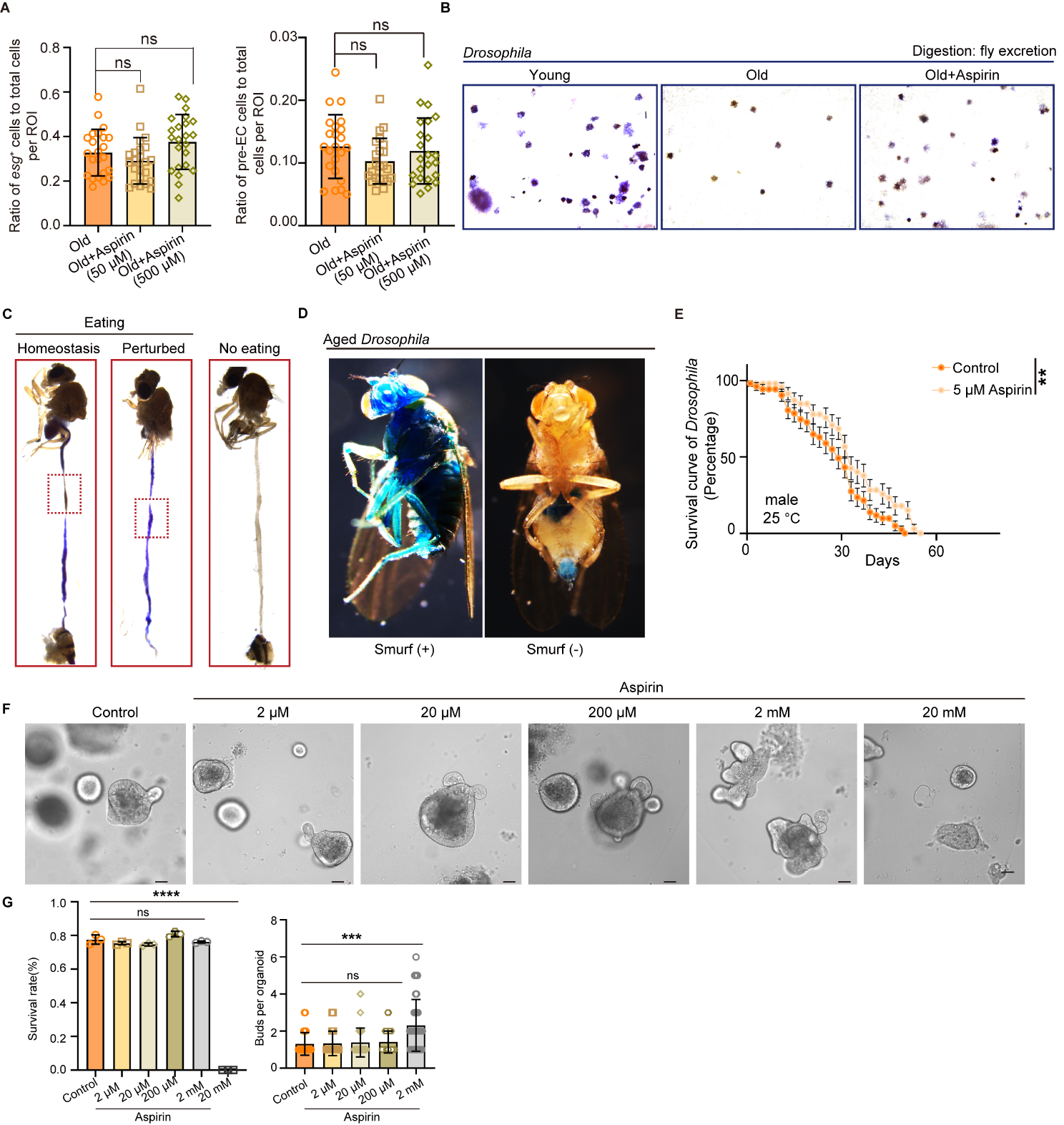


**Figure S1: Aspirin restrains age-associated ISC differentiation defect by enhancing the function of peroxisomes**

**(A)** Treatment of Drosophila with 50 µM or 500 µM aspirin failed to restrain the age-associated ISC differentiation defect.

**(B)** Excretion of *Drosophila* treated with Bromophenol blue.

**(C)** Representative images and quantification of *Drosophila* midguts treated with the pH indicator Bromophenol blue. There are three conditions: Homeostasis, a well-defined acidic (yellow colored) in copper cell region (CCR), and anterior midgut (AM) and posterior midgut (PM) is basic (blue colored); “Perturbed” the acidic region is lost and the whole gut is basic; “No eating”, no Bromophenol blue showed in the midguts.

**(D)** Representative images of “Smurf” flies after consuming a non-absorbed food dye.

**(E)** Aspirin treatment extended the lifespan of male *Drosophila.*

**(F-G)** Varying Aspirin concentrations (2 µM/20 µM/200 µM/2 mM/20 mM) on mouse intestinal organoids culturing.

#### Error bars represent SDs. Student’s t-tests, one-way ANOVA, Kruskal-Wallis test and log-rank test, *p < 0.05, **p < 0.01, ***p < 0.001, ****p < 0.0001, and NS (non-significant) represents p > 0.05. Underlying data and statistical analysis in S8 Data.
