# Supplementary material for: Aging-related peroxisomal dysregulation disrupts intestinal stem cell differentiation through alterations of very long-chain fatty acid oxidation
Source: PLoS Biol. 2025 Dec 19;23(12):e3003552. doi: 10.1371/journal.pbio.3003552 (PMC12716710; doi:10.1371/journal.pbio.3003552)
Supplement: S2 Fig — (A) The strategy used for constructing the endogenous Drosophila Pex5-Gal4 knock-in line. (B) Aspirin treatment also increased the expression of luciferase driving by Pex5-Gal4. (C) The strategy used for constructing the endogenous Drosophila Pex5-HA knock-in line. Error bars represent SDs. Student’s t tests, *p < 0.05, **p < 0.01, ***p < 0.001, ****p < 0.0001, and NS (non-significant) represent p > 0.05. Underlying data and statistical analysis in S9 Data. (DOCX) [file pbio.3003552.s002.docx]

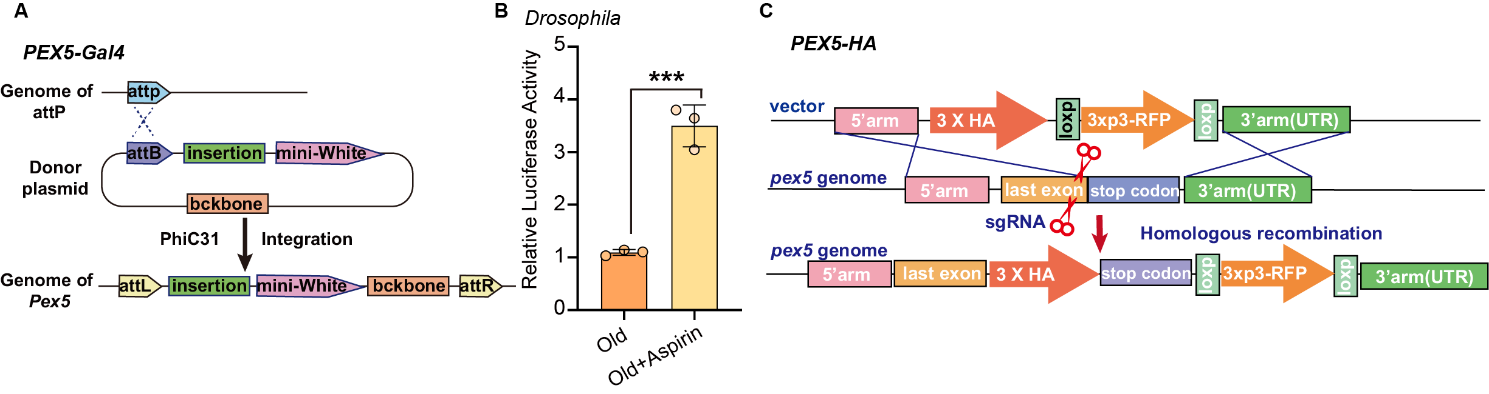


**Figure S2: PEX5-mediated peroxisomal matrix protein transportation is impaired in aged ISCs**

**(A)** The strategy used for constructing the endogenous *Drosophila* *Pex5-Gal4* knock-in line.

**(B)** Aspirin treatment also increased the expression of luciferase driving by *Pex5-Gal4.*

**(C)** The strategy used for constructing the endogenous *Drosophila* *Pex5-HA* knock-in line.

Error bars represent SDs. Student’s t tests, *p < 0.05, **p < 0.01, ***p < 0.001, ****p < 0.0001, and NS (non-significant) represents p > 0.05. Underlying data and statistical analysis in S9 Data.
