# Supplementary material for: Aging-related peroxisomal dysregulation disrupts intestinal stem cell differentiation through alterations of very long-chain fatty acid oxidation
Source: PLoS Biol. 2025 Dec 19;23(12):e3003552. doi: 10.1371/journal.pbio.3003552 (PMC12716710; doi:10.1371/journal.pbio.3003552)
Supplement: S3 Fig — (A) RT-qPCR analysis of Pex5 in mouse intestine organoids after transfection with siPex5 (n = 3 biologically independent mice per group). (B) Representative images showing the growth of organoids over 7 days. The organoids were transfected with control silencing RNA (siControl) and Pex5 silencing RNA (siPex5). (C) Quantification of organoids buds in each group. Error bars represent SDs. Scale bars represent 75 µm (A). Student’s t tests and one-way ANOVA, *p < 0.05, **p < 0.01, ***p < 0.001, ****p < 0.0001, and NS (non-significant) represents p > 0.05. Underlying data and statistical analysis in S10 Data. (DOCX) [file pbio.3003552.s003.docx]

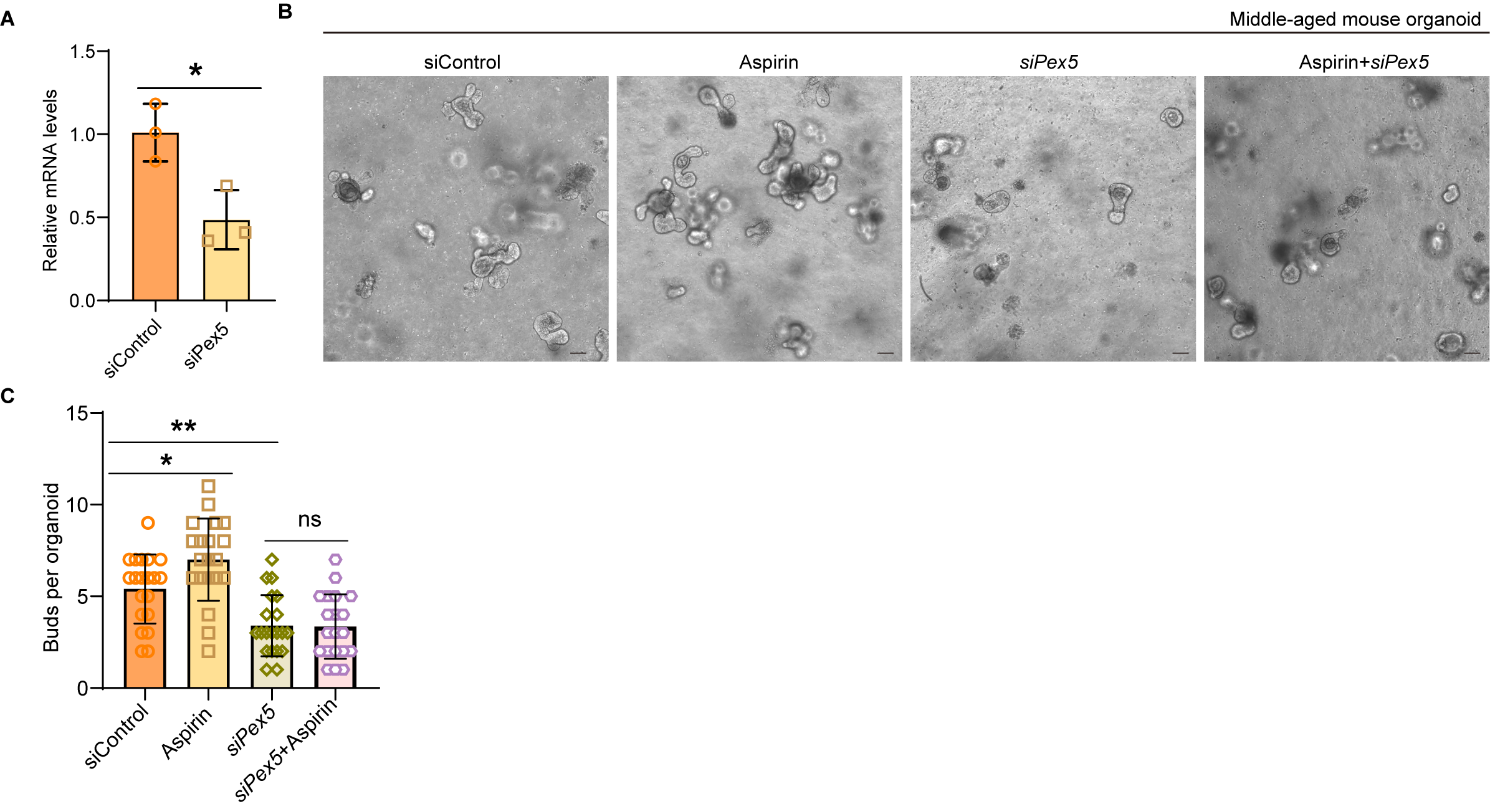


**Figure S3: Aspirin promotes aged ISC differentiation through the activation of PEX5-mediated PTS1 signaling**

**(A)** RT-qPCR analysis of *Pex5* in mouse intestine organoids after transfection with *siPex5* (n=3 biologically independent mice per group).

**(B)**Representative images showing the growth of organoids over 7 days. The organoids were transfected with control silencing RNA (siControl) and *Pex5* silencing RNA (*siPex5*).

**(C)** Quantification of organoids buds in each group.

Error bars represent SDs. Scale bars represent 75 µm (A). Student’s t-tests and one-way ANOVA, *p < 0.05, **p < 0.01, ***p < 0.001, ****p < 0.0001, and NS (non-significant) represents p > 0.05. Underlying data and statistical analysis in S10 Data.
