# Supplementary material for: Aging-related peroxisomal dysregulation disrupts intestinal stem cell differentiation through alterations of very long-chain fatty acid oxidation
Source: PLoS Biol. 2025 Dec 19;23(12):e3003552. doi: 10.1371/journal.pbio.3003552 (PMC12716710; doi:10.1371/journal.pbio.3003552)
Supplement: S4 Fig — (A) VLCFA detection in ISCs after Pex5 RNAi. (B–D) A significant reduce of SOX21A, 10× STAT-GFP, and RAB7-GFP expression in ISCs after Pex5 RNAi. Scale bars represent 10 µm (B, C, D). Error bars represent SDs. Student’s t-tests and Mann–Whitney test, *p < 0.05, **p < 0.01, ***p < 0.001, ****p < 0.0001, and NS (non-significant) represents p > 0.05. Underlying data and statistical analysis in S11 Data. (DOCX) [file pbio.3003552.s004.docx]

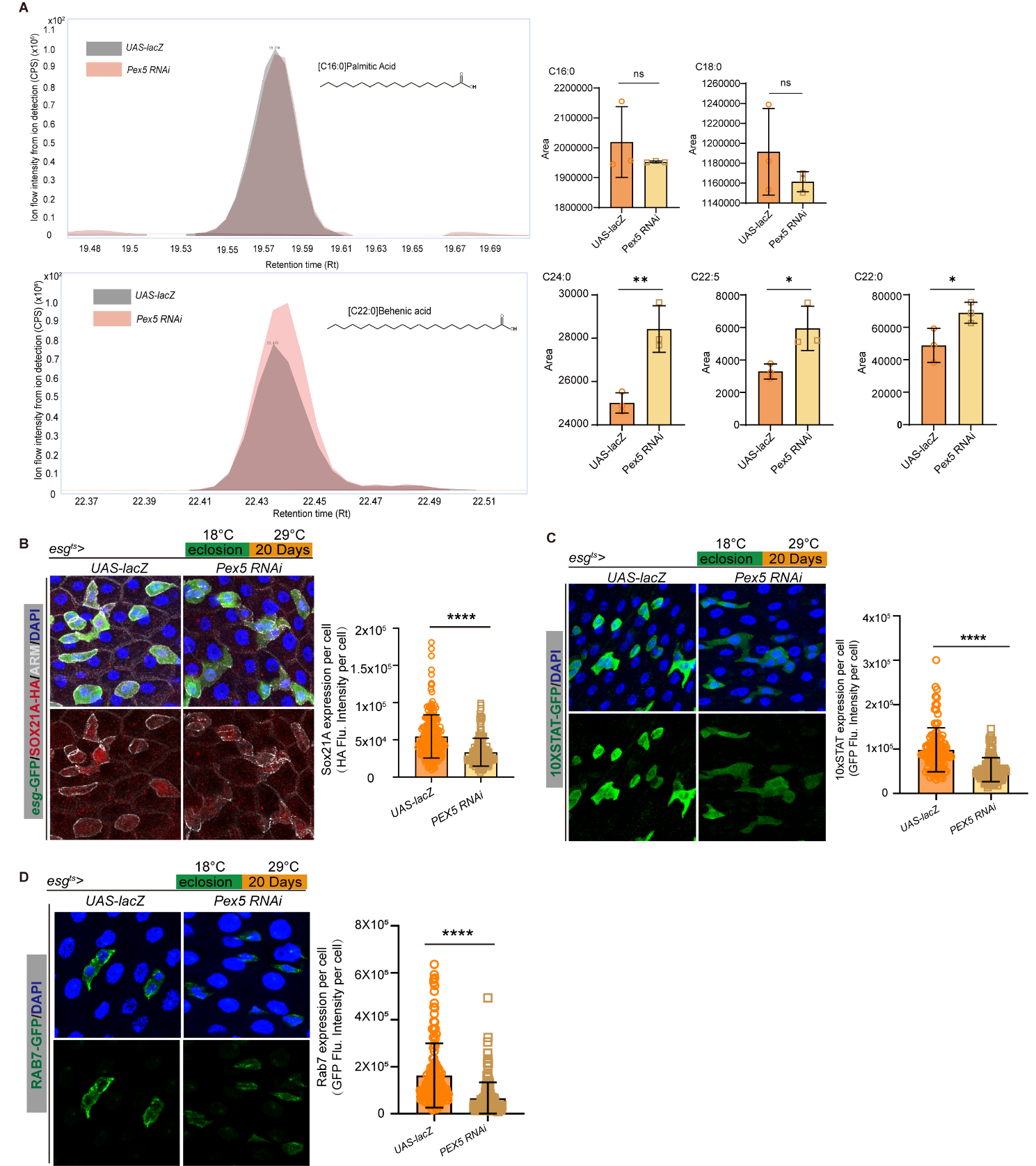


#### Figure S4: Impaired PEX5-mediated PTS1 signaling induce ISC mis-differentiation through modulation VLCFA metabolism

**(A)** VLCFA detection in ISCs after *Pex5* RNAi.

**(B-D)** A significantly reduce of SOX21A, 10X STAT-GFP and RAB7-GFP expression in ISCs after *Pex5* RNAi.

Scale bars represent 10 µm (B, C, D). Error bars represent SDs. Student’s t-tests and Mann Whitney test, *p < 0.05, **p < 0.01, ***p < 0.001, ****p < 0.0001, and NS (non-significant) represents p > 0.05. Underlying data and statistical analysis in S11 Data.
