# Supplementary material for: Aging-related peroxisomal dysregulation disrupts intestinal stem cell differentiation through alterations of very long-chain fatty acid oxidation
Source: PLoS Biol. 2025 Dec 19;23(12):e3003552. doi: 10.1371/journal.pbio.3003552 (PMC12716710; doi:10.1371/journal.pbio.3003552)
Supplement: S1 Text — (DOCX) [file pbio.3003552.s005.docx]

| **REAGENT or RESOURCE** | **SOURCE** | **IDENTIFIER** |
| --- | --- | --- |
| **Antibodies** | | |
| Chicken polyclonal anti-GFP | Abcam | Cat# ab13970  RRID: AB_300798 |
| anti-HA (C29F4) | Cell Signaling Technology | Cat# 3724  RRID: AB_1549585 |
| anti-Armadillo | DSHB | Cat# N2 7A1  RRID: AB_528089 |
| Monoclonal anti-mCherry | Invitrogen | Cat# M11217  RRID: AB_2536611 |
| Rabbit anti-Pdm1 | Gifted from XiaoHangYang | N/A |
| Anti-PMP70 | Sigma | Cat# SAB4200181  RRID: AB_10639362 |
| Anti-Catalase | GeneTex | Cat# GTX110704  RRID: AB_1949848 |
| Anti-alpha-Tubulin | Cell Signaling Technology | Cat# 2144  RRID: AB_2210548 |
| Anti-PEX5 | Invitrogen | Cat# PA5-58716  RRID: AB_2645411 |
| Anti-ACOX1 | Proteintech | Cat#10957-1-AP  RRID: AB_2221670 |
| Anti-PEX7 | Invitrogen | Cat# PA5-101744  RRID: AB_2851177 |
| HRP-mouse anti-rabbit | Jackson ImmunoResearch Labs | Cat# 211-032-171  RRID: AB_2339149 |
| HRP-Goat anti-mouse | Jackson ImmunoResearch Labs | Cat# 115-035-174  RRID: AB_2338512 |
| Goat anti-Mouse IgG (H+L) Cross-Adsorbed Secondary Antibody, Alexa Fluor™ 488 | Thermo Fisher Scientific | Cat# A-11001  RRID: AB_2534069 |
| Goat anti-Mouse IgG (H+L) Cross-Adsorbed Secondary Antibody, Alexa Fluor™ 568 | Thermo Fisher Scientific | Cat# A-11004  RRID: AB_2534072 |
| Goat anti-Mouse IgG (H+L) Cross-Adsorbed Secondary Antibody, Alexa Fluor™ 647 | Thermo Fisher Scientific | Cat# A-21235  RRID: AB_2535804 |
| Goat anti-Rabbit IgG (H+L) Highly Cross-Adsorbed Secondary Antibody, Alexa Fluor™ 488 | Thermo Fisher Scientific | Cat# A-11034  RRID: AB_2576217 |
| Goat anti-Rabbit IgG (H+L) Cross-Adsorbed Secondary Antibody, Alexa Fluor™ 568 | Thermo Fisher Scientific | Cat# A-11011  RRID: AB_143157 |
| Goat anti-Chicken IgY (H+L) Secondary Antibody, Alexa Fluor 488 | Thermo Fisher Scientific | Cat# A-11039  RRID: AB_2534096 |
| **Chemicals, peptides, and recombinant proteins** | | |
| DAPI (4’,6-Diamidino-2-phenylindole dihydrochloride) | Sigma-Aldrich | Cat# D8417 |
| Aspirin | Aladdin | Cat# A104180 |
| Behenic acid (BA) | Sigma | CAS# 112-85-6 |
| Bezafibrate | MCE | CAS# 41859-67-0 |
| Bromophenol blue sodium | Sigma | CAS# 34725-61-6 |
| Food Blue No.1 | Aladdin | CAS# 3844-45-9 |
| RIPA Lysis Buffer | Beyotime | Cat# P0013C |
| Protease Inhibitor Cocktail | MedChemExpress | Cat# HY-K0010 |
| PMSF | MedChemExpress | Cat# HY-B0496 |
| Loading buffer | Beyotime | Cat# P0015L |
| Electron Microscopy Sciences 8% Paraformaldehyde (formaldehyde) aqueous solution | Fisher Scientific | Cat# 5025997 |
| Trypsin-EDTA | Thermo Fisher Scientific | Cat# 15400-054 |
| Triton X-100 | Biofroxx | Cat# 9002-93-1 |
| Hematoxylin and eosin | Servicebio | Cat# G1003 |
| N2 | Thermo Fisher Scientific | Cat# 17502048 |
| B27 | Thermo Fisher Scientific | Cat# 17504044 |
| Wnt3a | MLB | Cat# J2-001 |
| Noggin | Peprotech | Cat# 250-38 |
| R-Spondin1 | Peprotech | Cat# 120-38 |
| N-acetyl-L-cysteine | Sigma | Cat# A7250 |
| Recombinant Mouse EGF Protein | Peprotech | Cat# 315-09 |
| SB202190 | Sigma | Cat# S7067 |
| Matrigel | BD bioscience | Cat# 356230 |
| Y-27632 | APExBIO | Cat# A3008 |
| A8301 | Tocris | Cat# 2939 |
| CHIR99021 | Med Chem Express | Cat# HY-10182 |
| nicotinamide | Aladdin | Cat#N108087 |
| Jagged-1 peptide | Anaspec | Cat#AS-61298 |
| **Critical commercial assays** | | |
| Firefly Luciferase Reporter Gene Assay Kit | Beyotime | Cat# RG005 |
| ECL chemiluminescence detection kit | Vazyme | Cat# E422-01 |
| BCA Protein Assay Kit | Beyotime | Cat# P0011 |
| Catalase Assay Kit | Beyotime | Cat# S0051 |
| RNA-easy Isolation Reagent | Vazyme | Cat# R701-01 |
| *Evo M-MLV* RT Mix Kit | Accurate Biology | Cat# AG11728 |
| ChamQ Universal SYBR qPCR Master Mix | Vazyme | Cat# Q712-02 |
| DAB Horseradish Peroxidase Color Development Kit | Beyotime | Cat# P0203 |
| Peroxisome Isolation Kit | Sigma | Cat# PEROX1 |
| **Deposited data** | | |
| Targeted quantitative GC-MS analysis of free fatty acids | This paper | Uploaded as Zip file to *The Plos Biology system* |
| RNA-seq data | This paper | SRA BioProject: PRJNA1169934 |
| Mass Spectrometry Proteomics data | This paper | iProX: PXD056574 |
| **Experimental models: Organisms/strains** | | |
| Drosophila lines |  |  |
| *w1118* | Bloomington Drosophila Stock Center | BDSC:3605; Flybase: FBst0003605 |
| Reporter: *esg-GFP* (Carnegie Protein Trap line esg^CB02017^) | from Allan Spradling | N/A |
| *esg^ts^-GAL4* line: *esg-GAL4, UAS-GFP, tub-Gal80^ts^/CyO* | from Benjamin Ohlstein | N/A |
| *UAS-LacZ* line: *w[*]; P{w[+mC]=UAS-lacZ.Exel}2* | Bloomington Drosophila Stock Center | BDSC:8529; Flybase: FBst0008529 |
| *UAS-LacZ* line: *w[*]; P{w[+mC]=UAS-lacZ.NZ}J312* | Bloomington Drosophila Stock Center | BDSC:3956; Flybase: FBst0003956 |
| *UAS-GFP-SKL* line: *y[1] w[*]; P{w[+mC]=UAS-GFP.SKL}2* | Bloomington Drosophila Stock Center | BDSC:28881; Flybase: FBst0028881 |
| *UAS-PEX5-HA* | This paper | N/A |
| *UAS-Sox21a* line*:* *w*; P{UAS-Sox21a.C}8* | Bloomington Drosophila Stock Center | BDSC:68156 Flybase: FBst0068156 |
| *UAS-Rab7-CA* line*:* *P{w[+mC]=UASp-YFP.Rab7.Q67L}7, y[1] w[*]* | Bloomington Drosophila Stock Center | BDSC: 50785 Flybase: FBst0050785 |
| *UAS-Luciferase:* *y[1] v[1]; P{y[+t7.7] v[+t1.8]=UAS-LUC.VALIUM10}attP2* | Bloomington Drosophila Stock Center | BDSC: 35788  Flybase: FBst0035788 |
| Reporter: *SOX21A-HA line:* | This paper | N/A |
| *Elovl RNAi: y[1] sc[*] v[1] sev[21]; P{y[+t7.7] v[+t1.8]=TRiP.HMC03112}attP2* | Bloomington Drosophila Stock Center | BDSC: 50710 Flybase: FBst0050710 |
| *ACOX1 RNAi: y[1] sc[*] v[1] sev[21]; P{y[+t7.7] v[+t1.8]=TOE.GS00506}attP40* | Bloomington Drosophila Stock Center | BDSC:68109 Flybase: FBst0068109 |
| *PEX5 RNAi* | TsingHua Fly Center | THFC: TH02247.N |
| *esg-GAL4* | from Allan Spradling | N/A |
| *PEX5-HA* | This paper | N/A |
| *Pex10-mCherry* | This paper | N/A |
| *PEX5-GAL4* | This paper | N/A |
| *Reporter 10XStat-GFP: w[1118]; P{w[+mC]=10XStat92E-DGFP}3/TM6C, Sb[1] Tb[1]* | Bloomington Drosophila Stock Center | BDSC: 26200 Flybase: FBst0026200 |
| *Reporter GFP-CAT: w[*]; P{w[+mC]=*  *PTT-GC}Cat[CC00907]/TM3, Ser[1] Sb[1]* | Bloomington Drosophila Stock Center | BDSC: 51546 FlyBase:  FBst0051546 |
| *UAS-Rab7-GFP line: w[*]; P{w[+mC]=UAS-Rab7.GFP}3* | Bloomington Drosophila Stock Center | BDSC: 42706 FlyBase: FBst0042706 |
| *Acox1-Flag* | This paper | N/A |
| Mouse line |  |  |
| C57BL/6 J male mice | GemPharmatech (Nanjing, China) | N/A |
| C57BL/6 J male mice | Aniphe Biolaboratory Inc | N/A |
| **Oligonucleotides** | | |
| Primers for real time-qPCR, see Table S2 | This paper | N/A |
| Primers for transgenic fly, see Table S2 | This paper | N/A |
| **Recombinant DNA** | | |
| Vector: PMD18T | This paper | N/A |
| Vector: PCR8 | This paper | N/A |
| Vector: attB | This paper | N/A |
| Vector: pEntry | This paper | N/A |
| Vector: pTW | from Allan Spradling | N/A |
| Vector: pcDNA3.1-FLAG-HA | Youbio Biological Technology Co., Ltd. | Cat# VT8014 |
| **Software and algorithms** | | |
| ImageJ | ImageJ | https://imagej.nih.gov/ij/ |
| Prism 7.0 | GraphPad | https://www.graphpad.com/ |
| LAS X | Leica | N/A |
| **Others** | | |
| TCS-SP8 confocal microscope | Leica | Leica TCS-SP8 |
| Upright Microscopes | Leica | Leica DM6 B |
| Fluorescence stereo microscopes | Leica | Leica M205 FA |
| PCR | Bio-Rad | C1000 Touch Thermal Cycler |
| Multifunctional microplate reader | BMG LABTECH | CLARIOstar |
