# Supplementary figures and images for: Aging-related peroxisomal dysregulation disrupts intestinal stem cell differentiation through alterations of very long-chain fatty acid oxidation
Source: PLoS Biol. 2025 Dec 19;23(12):e3003552. doi: 10.1371/journal.pbio.3003552 (PMC12716710; doi:10.1371/journal.pbio.3003552)

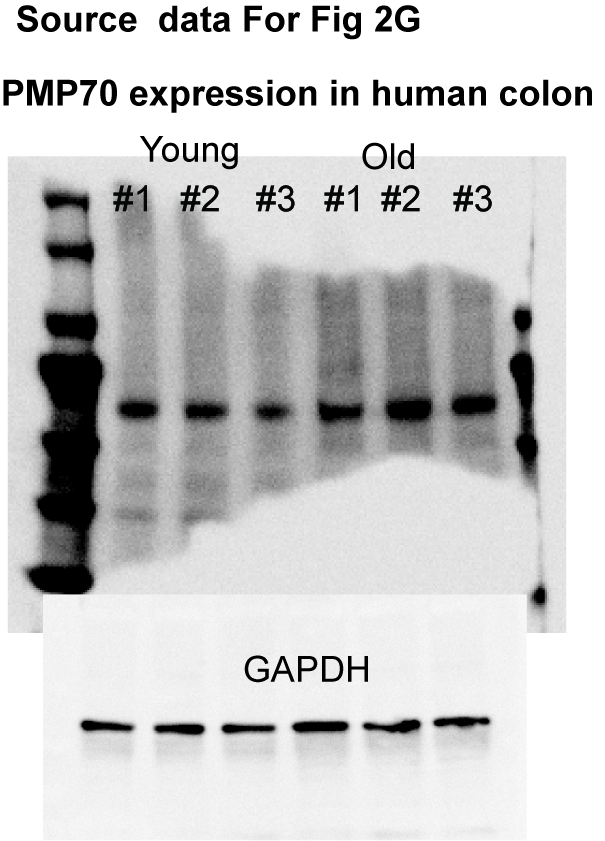

Supplement: S1 Raw Images — (TIF) [file pbio.3003552.s020.tif]

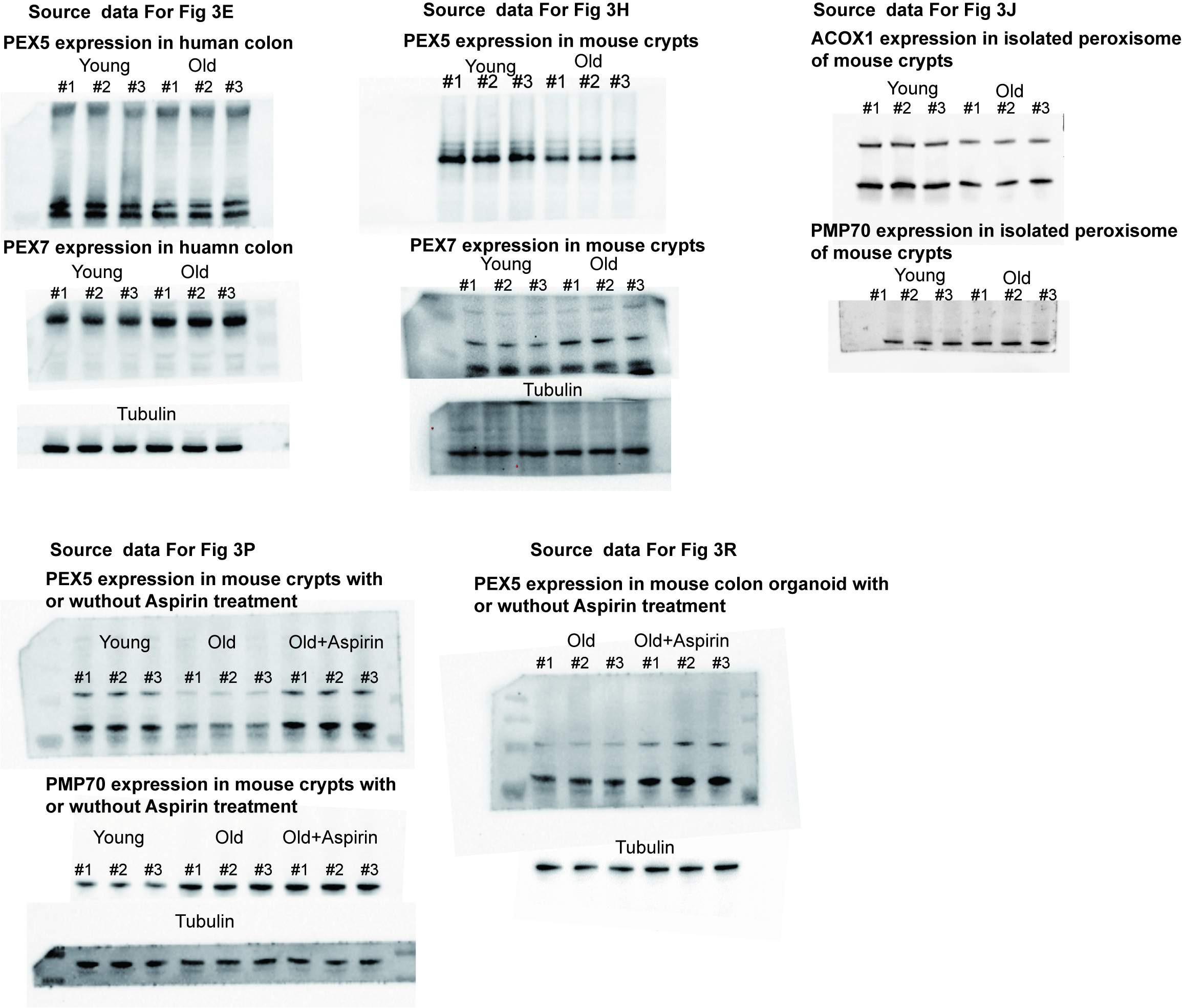

Supplement: S2 Raw Images — (TIF) [file pbio.3003552.s021.tif]
