# Supplementary material for: Aging-related peroxisomal dysregulation disrupts intestinal stem cell differentiation through alterations of very long-chain fatty acid oxidation
Source: PLoS Biol. 2025 Dec 19;23(12):e3003552. doi: 10.1371/journal.pbio.3003552 (PMC12716710; doi:10.1371/journal.pbio.3003552)
Supplement: Attachment — Submitted filename: Response Letter to Reviewers.docx [file pbio.3003552.s024.docx]

**Reply to Reviewer：**

In their manuscript, Guo et al. reveal how aging impairs intestinal stem cell (ISC) function through peroxisomal dysfunction and dysregulated lipid metabolism. Using Drosophila and mouse intestinal organoids, the study demonstrates that reduced PEX5 expression in aged ISCs leads to impaired import of peroxisomal matrix proteins and the accumulation of very long-chain fatty acids (VLCFAs). The authors identify RAB7-dependent endosome maturation and SOX21A as downstream regulators controlling ISC differentiation. Notably, Aspirin restores ISC lineage fidelity by enhancing PEX5-mediated peroxisomal β-oxidation of VLCFAs, suggesting a potential therapeutic strategy to counteract age-related intestinal decline. Although the novelty of this work is partially limited by previous studies—such as the reported role of Aspirin in regulating intestinal stem cell (ISC) differentiation via Wnt signaling inhibition (PMID: 32971322), the established link between peroxisomal import dysfunction and aging (PMID: 32523050), and the documented function of very-long-chain fatty acids (VLCFAs) as signaling molecules in peroxisome homeostasis and intestinal repair (PMID: 32243783)—this study consolidates a critical pathway governing aging-associated ISC differentiation. Nevertheless, several key concerns must be addressed before the manuscript can be considered for publication, as detailed below:

*Response:*

*We genuinely value the thorough evaluation conducted by the reviewer on our manuscript and the insightful comments provided. In response to the reviewer's concerns, we have responded all the questions raised by offering additional results as below. We believe the revision we made will address all concerns of the reviewer.* *Furthermore, we are committed to undertaking additional experiments to address any remaining issues.*

1. Since this study used both drosophila and mammalian systems, the authors should clarify whether the claimed pathway is conserved or not, e.g. whether *drosophila* has PTS2? Is there any difference between the preEC accumulation in old *drosophila* and budding defect in mouse intestine organoids?

*Response:*

(1) Since this study used both drosophila and mammalian systems, the authors should clarify whether the claimed pathway is conserved or not, e.g. whether *drosophila* has PTS2?

*Thank you for your feedback. It is crucial to confirm that whether peroxisomal matrix protein importing is conserved between Drosophila and mammals. In mammals, it has been reported that peroxisomal matrix proteins have either a C-terminal peroxisomal targeting signal 1 (PTS1) or an N-terminal PTS2. These proteins are recognized by specific receptors, Pex5, for PTS1 and Pex7 for PTS2 and then imported into peroxisome (Di Cara, Savary, Kovacs, Kim, & Rachubinski, 2023). However, although Drosophila encodes a Pex7 homologue as mammals, a functional* *PTS2 system remains to be identified (Di Cara, Rachubinski, & Simmonds, 2019). But it is undeniable that most peroxisomal matrix proteins rely on PEX5-mediated PTS1 transportation, with only a few matrix proteins rely on PTS2 in mammalian systems (Gould, Krisans, Keller, & Subramani, 1990; Walker, Pomatto, Tripathi, & Davies, 2018). Therefore, PEX5-mediated PTS1 pathway is highly conserved both in Drosophila and mammals. Besides, we have included the explain in the revised manuscript. Once again, thanks for your suggestion.*

(2) Is there any difference between the preEC accumulation in old *drosophila* and budding defect in mouse intestine organoids?

*Thank you for your feedback. In fact, both the pre-enterocyte (preEC) accumulation in aged Drosophila and the budding defect in aged mouse intestinal organoids reflect a decline in the differentiation capacity of aged ISCs. Because that: (1) many studies have reported that the uncontrolled differentiation of ISCs in the aged Drosophila intestine leads to the accumulation of progenitor cells enteroblasts (EBs, esg^+^ cell) and pre-enterocyte (preEC) (Luo et al., 2025; Yan et al., 2022; Zhang et al., 2023). (2) Organoid budding mirrors de-novo crypt formation in vivo: it results from ISCs undergoing self-renewal, symmetric/asymmetric division, and differentiation into progenitors. When ISC differentiation is impaired, crypt initiation and expansion stall, then budding defects producing. And it has been reported that crypts from aged mice (>24 months) exhibit significantly impaired budding compared to those from young mice (3-9 months) (Pentinmikko et al., 2019).*

*However, there are also several differences between them. For instance, Paneth cell-mediated stem cell niche effects intestinal organoid formation, which is absent in the Drosophila midgut. Then, in this study, we found that aging reduces PEX5 in ISCs, causing peroxisomal dysfunction and very long-chain fatty acids (VLCFAs) accumulation that impairs ISC differentiation. This mechanism on effect aged ISC differentiation is highly conserved in both Drosophila and mammalian systems.*

2. The study primarily focuses on Aspirin's role in improving ISC differentiation via the PEX5 pathway in Drosophila models but lacks validation in mammalian systems (e.g., mouse or human cells). This limits the generalizability and clinical relevance of the findings.

*Response:*

*Thank you for your feedback. We have addressed your concerns as follows.*

*(1) Firstly, in our initial manuscript we have demonstrated that Aspirin's role in improving ISC differentiation via the PEX5 pathway in Drosophila models. As knockdown Pex5 in ISCs resulted in a significant accumulation of preEC, which mimics the phenotype of aged midguts in Drosophila (termed as mimic aging; Fig. 4F, 4G), where are Aspirin did not rescue this phenotype (Fig. 4F, 4G).*

*(2) Besides, in our initial manuscript we also have analyzed the expression of PEX5 in the aged mouse colon tissue and colon organoid treated with Aspirin. The results showed a significant increase in PEX5 expression, confirming that Aspirin improve ISC differentiation by promoting PEX5 expression in mammalian systems (Fig.3O-3T).*

*(3) In addition, to further confirm that Aspirin enhances ISC differentiation through PEX5 in mammalian systems, we silenced Pex5 in middle aged mouse colon organoids. Then we found that Pex5 knockdown markedly reduced mouse organoid budding, and subsequent Aspirin treatment failed to restore its metrics (Fig. S3A-C). Therefore, these data indicate that Aspirin’s pro-differentiation effect is strictly PEX5-dependent.*

*Once again, thanks for your suggestion.*

*Fig. 4F, 4G*

*Fig.3O-3T*

*Fig.S3A-S3C*

3. While the data suggest a correlation between decreased PEX5 levels and VLCFA accumulation, direct evidence (e.g., PEX5 overexpression directly reducing VLCFA levels) is lacking.

*Response:*

*We appreciate your valuable suggestions. To explore the relationship between decreased PEX5 levels and VLCFA accumulation, we have performed the following experiment:*

*VLCFA Detection after Pex5 RNAi in flies: we used ISC-specific drivers (esg^ts^-Gal4) to knock down Pex5 (esg^ts^>Pex5 RNAi) in ISCs cells, and* *liquid chromatography-mass spectrometry (LC-MS) was used to analyze free fatty acids levels. Then, we found that free VLCFAs (such as* *Behenic acids (C22:0)、Lignoceric acids (C24:0)) were clearly increased, and other long chain fatty acids (such as* *palmitic acids (C16:0)、Stearic acids (C:18)) have no obvious changing, which demonstrated that decreased PEX5 levels lead to VLCFA accumulation (Fig. S4A).*

*Therefore, this is the direct evidence of PEX5's role in VLCFA metabolism and clarify the link between PEX5 decrease and VLCFAs accumulation.*

*Fig. S4A*

4. There is a significant discrepancy in the Aspirin concentrations used between Drosophila (5 µM) and mouse intestinal organoids (2 mM). The rationale for this difference should be clarified.

*Response:*

*Thank you for your feedback. The Aspirin concentrations used in Drosophila (5 µM) and mouse intestinal organoids (2 mM) are based on the following considerations:*

*1.* ***Drosophila****: In our study，we explored the effects of varying Aspirin concentrations (5 µM, 50 µM, and 500 µM) on aged flies. And revealed that 5 µM Aspirin was the most effective concentration, as it significantly diminished aged ISCs differentiation defect (Fig. 1C-1E, and S1A) and promoted longevity (Fig. 1I).*

*2.* ***Mouse Intestinal Organoids****: We have explored* *varying Aspirin concentrations (2 µM, 20 µM, 200 µM, 2 mM, and 20 mM) on mouse intestinal organoids culture (Fig.S1F, S1G). And found that 2 mM Aspirin is the optimal concentration for aged mouse intestinal organoids forming and budding, which was consistent with reported (Dunbar et al., 2021).*

*3.* ***Species Differences:*** *The concentration difference reflects the distinct physiological and metabolic characteristics of Drosophila and mice. The lower concentration in Drosophila is due to its more sensitivity in Aspirin, while the higher concentration in mouse organoids accounts for their more complex metabolic environment.*

*Once again, thanks for your suggestion. We have included this section in the Discussion of revised manuscript.*

*Fig. 1C-E, 1I and S1A*

*Fig. S1F, S1G*

5. Why does only 5 µM Aspirin enhance fly survival (Fig. 1i), while higher concentrations (50 µM, 500 µM) show no effect? A dose-response curve or mechanistic explanation is needed to address this observation.

*Response:*

*We appreciate the reviewer’s insightful question regarding the dose-dependent effects of Aspirin in our study. Indeed, our data show that 5 µM Aspirin significantly enhances fly survival, while higher concentrations (50 µM, 500 µM) do not exhibit a similar protective effect (Fig. 1I). This observation aligns with well-documented biphasic or hormetic responses to Aspirin and other nonsteroidal anti-inflammatory drugs (NSAIDs), where low and high doses can exert distinct biological effects. Many studies have shown that low-dose Aspirin positively contribute to intestinal homeostasis in Drosophila (Zhu et al., 2021), acts as an epigenetic suppressor in human intestinal aging(Noreen, Chaber-Ciopinska, Regula, Schar, & Truninger, 2020), and reduces the incidence of colorectal cancer(Drew, Cao, & Chan, 2016). However, high-dose Aspirin can cause gastrointestinal side effects, such as ulceration and bleeding (Zhu, Wang, Zhao, Wang, & Sang, 2017).*

*Similarly, we also found a side effects of high-dose Aspirin (50 µM, 500 µM) exist in fruit flies, as manifest as disruption of gut epithelial integrity (shown as Arm staining) as below.*

*Fig. 1I*

*A side effects of high-dose Aspirin (50 µM, 500 µM) exist in fruit flies, as manifest as disruption of gut epithelial integrity.*

6. In Fig. 2g, the increase in PMP70 appears marginal. Grayscale quantification (e.g., band intensity analysis) should be provided to confirm the significance of the results.

*Response:*

*Thank you for your feedback. We acknowledge your concern regarding the marginal increase in PMP70 observed in Fig. 2g. To address this, we have reperform this western Blots and re-quantify its grayscale to confirm that the PMP70 levels is increased in aged human colon. We appreciate your suggestion and have incorporate this analysis into the revised manuscript as Fig 2G.*

*Fig. 2G*

7. In Fig. 3m, the method for quantifying GFP-SKL fluorescence intensity (e.g., normalization per peroxisome) is not described, making it difficult to interpret the data.

*Response:*

*Thank you for your feedback. We appreciate your concern regarding the quantification of GFP-SKL fluorescence intensity in Fig. 3M. To clarify, the fluorescence intensity was quantified using the fluorescence analysis tool in Leica confocal software. For each ISC cell, the total fluorescence intensity of GFP-SKL was measured, and the number of GFP-SKL puncta within the cell was counted. Then, the average fluorescence intensity per GFP-SKL punctum was calculated by dividing the total fluorescence intensity by the number of GFP-SKL puncta. In addition, we have included this detailed description of the quantification method in the revised manuscript to ensure clarity and reproducibility of the data. Thank you for your suggestion.*

8. In Fig. 1i and 5g, the number of experimental replicates and sample size per group are not stated, which compromises the assessment of statistical power.

*Response:*

*Thank you for your feedback. We appreciate your concern regarding the experimental replicates and sample size in the lifespan experiments presented in Fig. 1Iand 5G. In the original manuscript, we only shown one representative experiment with 50 flies per group. To address your concern and ensure our findings, we have included other two additional replicates as below. Besides, we also used male Drosophila for lifespan analysis, with Aspirin treatment and overexpression or knock down Pex5 specific in ISCs. As a results, we found it is similar with the results of female Drosophila, as Aspirin treatment and overexpression PEX5 increased its lifespan, while knock down Pex5 decreased. Therefore, we sure these results will enhance the conclusion that Aspirin and PEX5 overexpression can extent the lifespan of flies, both in female and male. Once again, thank you for your suggestion.*

*Lifespan analysis* *used female Drosophila*

*Lifespan analysis* *used male Drosophila*

**Academic Editor’s comments**

This manuscript investigates the mechanisms of intestinal stem cell (ISC) aging using Drosophila and mouse colon organoid models. The authors present evidence suggesting that age-related decline in PEX5 expression compromises peroxisomal matrix protein import in ISCs, which is associated with peroxisomal dysfunction and very long-chain fatty acid (VLCFA) accumulation. Their findings indicate that elevated VLCFA levels may disrupt ISC differentiation by interfering with RAB7-dependent late endosomal maturation and JAK-STAT-SOX21A signaling. The authors also report that Aspirin treatment appears to restore proper ISC lineage commitment by promoting PEX5-mediated peroxisomal β-oxidation of VLCFAs. While these results point to peroxisomal function and lipid metabolism as potential regulators of ISC aging, the current manuscript would benefit from additional experimental evidence to strengthen the proposed causal relationships and fully support the conclusions.

Substantial revision is recommended to address these limitations.

*Response:*

*We appreciate that the Academic Editor gives* *positive comments on our study.* *In order address the issues mentioned by the Academic Editor, we have provided a detailed explanation of these comments and outlined our experimental results* *in the current revised version.*

**Major Comments:**

1. The authors demonstrate that Aspirin treatment can restore the diminished PEX5 levels observed in aged mice and colon organoids. They also show that aged mouse colon tissues exhibit elevated VLCFA concentrations, which are reduced following Aspirin administration. However, the mechanistic connection between PEX5 and VLCFA metabolism requires more robust investigation through genetic approaches. Additional experiments involving PEX5 manipulation would strengthen the proposed relationship between these two factors.

*Response:*

*We appreciate your valuable suggestions. To explore the relationship between decreased PEX5 levels and VLCFA accumulation, we have performed the following experiment:*

***VLCFA Detection after Pex5 RNAi in flies:*** *we used ISC-specific drivers (esg^ts^-Gal4) to knock down Pex5 (esg^ts^>Pex5 RNAi) in ISCs cells, and* *liquid chromatography-mass spectrometry (LC-MS) was used to analyze free fatty acids levels. As a result, we found that free VLCFAs (such as Behenic acids (C22:0)、Lignoceric acids (C24:0)) were clearly increased, and other shorter fatty acids (such as palmitic acids (C16:0)、Stearic acids (C:18)) have no obvious changing, which demonstrated that decreased PEX5 levels has a strongly association with VLCFA accumulation (Fig. S4A).*

*Therefore, this is the direct evidence of PEX5's role in VLCFA metabolism and clarify the link between PEX5 decrease and VLCFAs accumulation.*

*Fig. S4A*

2. While the study demonstrates reduced PEX5 expression in aged intestinal stem cells concurrent with peroxisomal dysfunction, the causal relationship between these phenomena requires further experimental validation. Additional mechanistic studies would be valuable to establish whether PEX5 downregulation directly leads to the observed peroxisomal impairment.

*Response:*

*Thank you for your insightful questions.* *In initial version, we partially demonstrated that PEX5 downregulation impairs the function of peroxisomal matrix proteins importing. Specifically, by knocking down Pex5 in the ISC of young Drosophila (esg > Pex5 RNAi), we observed that peroxisomes were uniformly dispersed throughout the entire cell without aggregation (Fig. 4A).*

*In the revised version, to further confirm that PEX5 downregulation directly leads to peroxisomal impairment, we knocked down Pex5 in the ISC, and performed additional experiments as below：*

*(1)* ***Catalase endogenous expression:*** *Catalase is the most highly expressed and well-characterized* *peroxisomal antioxidant enzyme, which is transported into peroxisome by PEX5 receptor, responsible for scavenging harmful H₂O₂ and protecting cells from oxidative stress. To explore* *whether knocked down Pex5 affect the expression of Catalase, a* *Catalase-GFP Drosophila reporter line (CAT-GFP) was exploited. As a result, after knockdown of PEX5 in ISCs, a reduce of the Catalase endogenous expression was found (Fig. 4B, 4C), which indicated that knockdown of PEX5 lead to the impairment of peroxisomal antioxidant function.*

*(2)* ***ACOX1 endogenous expression:*** *Peroxisomal ACOX1 (acyl-CoA oxidase 1) is the first and* *rate-limiting enzyme in peroxisome β-oxidation of VLCFAs, which is also transported into peroxisome by PEX5 receptor. To trace the endogenous expression of ACOX1 after knock down of Pex5, we generated an endogenous peroxisome Drosophila reporter line ACOX1-Flag, using the CRISPR-Cas9 knock-in system. As a results, the expression of ACOX1 in ISCs was decreased after Pex5 RNAi (Fig. 4D, 4E), which indicated that knockdown of PEX5 lead to the impairment of peroxisomal VLCFAs β-oxidation function.*

*(3)* ***VLCFA Detection:*** *Peroxisome are responsible for the β-oxidation of VLCFAs (with a chain length of ≥20 carbon).Liquid chromatography-mass spectrometry (LC-MS) was used to analyze free fatty acids levels used the Drosophila of Pex5 RNAi (esg > Pex5 RNAi). As a result, we found that free VLCFAs (such as Behenic acids (C22:0)、Lignoceric acids (C24:0)) were clearly increased, and other shorter fatty acids (such as palmitic acids (C16:0)、Stearic acids (C:18)) have no obvious changing, which demonstrated that decreased PEX5 levels has a strongly association with VLCFA accumulation (Fig. S4A).*

*Thus, all these data were consistent with our conclusion that PEX5 downregulation leads to the impairment of peroxisome function.*

*Fig. 4A*

*Fig. 4B-4E*

*Fig. S4A*

3. The manuscript proposes that peroxisomal β-oxidation of VLCFAs, regulated by PEX5, influences ISC differentiation through modulation of RAB7-dependent late endosome maturation and the JAK-STAT-SOX21A pathway. However, this mechanistic framework lacks sufficient supporting evidence or relevant citations from the literature. Additional experimental data or references are needed to establish the proposed connection between peroxisomal function and these signaling pathways in ISC differentiation.

*Response:*

*Thanks for your suggestion. We apologize for not detailly verify the relationship of peroxisomal function with RAB7-dependent late endosome maturation and JAK-STAT-SOX21A pathway in aged ISC differentiation.*

*a. In fact, in our previous study, we have demonstrated that they are the downstream of peroxisome in control ISC differentiation during intestinal epithelial repair (Du, Xiong, et al., 2020). In that study, we demonstrated that peroxisomes are essential for ISC production of differentiated ECs during midgut regeneration. Two peroxisomal mutant flies Pex2-null and Pex10-null were used, which result in the formation of “ghost” peroxisomes (the structure of peroxisome is still maintained, but the function of peroxisome is disrupted). Forced expression of Sox21a, or a constitutively active version of JAK (hopscotch [hop]) (UAS-hop^TUM^) or RAB7 (RAB7-CA) partially rescued the differentiation defect of ISC caused by Pex2-null and Pex10-null. Therefore, we demonstrated that peroxisomes by enhancing RAB7-dependent late endosome maturation and then modulating of JAK and STAT-SOX21A signaling, which in turn promoting ISC differentiation during intestinal epithelial repair.*

*b. In the current study, we found that the decreased expression of PEX5 in aged ISCs lead to ISCs mis-differentiation.* *To further confirm RAB7-dependent late endosome maturation and JAK-STAT-SOX21A signaling, are regulated by PEX5 in induce aged ISC differentiation, ISCs specifically Pex5 RNAi Drosophila was employed. As a result, we found that a significant reduce in SOX21A, 10XSTAT-GFP, and RAB7-GFP expression in ISCs after Pex5 RNAi* *(Fig. S4B-D). In addition, we also have demonstrated that forced expression SOX21A or the constitutively active version of RAB7 (RAB7-CA) significantly restored the differentiation defect of ISCs in Pex5 RNAi, respectively (Fig. 7M-N). Therefore, these findings confirm that the JAK-STAT-SOX21A and RAB7-dependent late endosome maturation pathway is the downstream of peroxisomal in control aged ISC differentiation.*

*Peroxisome control ISC differentiation via RAB7-dependent late endosome maturation and JAK-STAT-SOX21A pathway during intestinal epithelial repair (Du et al 2020)*

*Fig. S7B-D*

*Fig. 7M-N*

4. Further investigation is warranted to determine whether upregulation of SOX21A or RAB7 could rescue the differentiation defects observed in aged intestinal stem cells. This experimental approach would help establish the functional significance of these factors in age-related ISC differentiation impairment.

*Response:*

*We appreciate your comments and suggestions. We agree the experiments about whether upregulation of SOX21A or RAB7 could rescue the differentiation defects observed in aged ISCs are necessary to perform. In fact, in our recently published study we have found a reduced RAB7 expression in aged human duodenum (Yu et al., 2024) and ISCs of aged Drosophila (Du, Qiao, et al., 2020). We have demonstrated that forced expression of a constitutively active version of RAB7 (RAB7-CA) or overexpressed SOX21A can rescue aged ISC differentiation defects as below (Yu et al., 2024). In addition, in the current study we also have demonstrated that forced expression them significantly restored the differentiation defect of ISCs caused by Pex5 RNAi (Fig. 7M-N). Once again, we appreciate your valuable suggestions.*

*Fig.5 h in Yu et al 2024*

*Fig. 7M-N*

5. The quality of the Western blot is poor, the data is overexposed, and the changes mentioned by the authors cannot be discerned, e.g., Figure 2G, 3E, 3J. This insufficient data quality undermines the conclusions drawn from these figures.

*Response:*

*Thank you for highlighting the deficiencies in the Western blot data. We apologize for the overexposed or low-quality images in Fig. 2G, 3E, and 3J. We have now repeated the blots for Fig. 2G and 3E, optimized the exposure for Fig. 3J, and performed quantitative analysis for all Western blots. The revised blot images and their statistics are presented in the updated manuscript.*

*Fig. 2G, 3E, 3J, 3P and 3R*

6. When describing the results of Western blot and certain immunofluorescence images (e.g., Figure 2O), the manuscript uses qualitative terms like "increased" or "decreased" without providing statistical analysis. It is crucial to include statistical results to support these qualitative descriptions.

*Response:*

*Thank you for raising this important point. We fully agree that incorporating statistical analysis is essential to substantiate the results of our Western blot and immunofluorescence data. In response, we have re-run the Western Blots for Fig. 2G, 3E, and conducted a thorough statistical analysis of both the Western blot and immunofluorescence data. Once again, we appreciate your valuable suggestions.*

*Fig. 2G, 3E, 3J, 3P, 3R and 2P*

7. The manuscript lacks data explaining how Aspirin was identified as a potential candidate. It is crucial to provide evidence or data from the screening process that led to the selection of Aspirin for further investigation. Please include detailed information on the screening process, such as the criteria used, the number of compounds tested, and the specific data that highlighted Aspirin as a promising candidate.

*Response:*

*Thank you for your insightful comment regarding the identification of Aspirin as a potential candidate. In our preliminary studies,* *we conducted a comprehensive screening of 31 small-molecule anti-aging compounds across both Drosophila and mouse intestinal organoid models. The compounds were evaluated based on their ability to reduce the proportion of esg-GFP and Pre-EC cells in old flies or to enhance the budding and formation rates in aged mouse intestinal organoids.* *Through this rigorous screening process, Aspirin emerged as a promising candidate alongside other compounds—including* *Quercetin (Yan et al., 2022), Lipoic acid (Du, Qiao, et al., 2020), Taurine (Du et al., 2021)，Kaempferol (Zhao et al., 2024) and Asparagine (Luo et al., 2025)—that have been previously reported by us. Once again, we appreciate your valuable suggestions, and we have included this section into in the revised manuscript.*

8. In Figure 3S, mass spectrometry analysis of peroxisomes was performed using colon tissues from aged mice, both with and without Aspirin treatment. However, the data displayed considerable heterogeneity between individual samples, and there are no annotations in the figure legend to clarify these observations. To enhance the reliability and interpretability of these data, I recommend performing unsupervised principal component analysis (PCA), which could help account for this variability and further strengthen the conclusions.

*Response:*

*Thank you for your suggestion regarding the analysis of our mass spectrometry data. In response to your comment, we have added unsupervised principal component analysis (PCA) analysis of the protein mass spectrometry data from peroxisomes of aged mice, both with and without Aspirin treatment as Fig. 3S. We appreciate your guidance in improving the quality of our manuscript.*

*Fig.3S*

9. In Figure 1J, the concentration of Aspirin used to treat mouse intestinal organoids (2 mM) is significantly higher than the 5 µM used in Drosophila. This discrepancy needs to be addressed and explained. Additionally, it would be valuable to investigate whether there is a dose-response effect of Aspirin on mouse intestinal organoids.

*Response:*

*Thank you for your feedback. The Aspirin concentrations used in Drosophila (5 µM) and mouse intestinal organoids (2 mM) are based on the following considerations:*

*1.* ***Drosophila****: In our study，we explored the effects of varying Aspirin concentrations (5 µM, 50 µM, and 500 µM) on aged flies. And revealed that 5 µM Aspirin was the most effective concentration, as it significantly diminished aged ISCs differentiation defects (Fig. 1C-E and Fig. S1A) and promoted longevity (Fig. 1I).*

*2.* ***Mouse Intestinal Organoids****: We have explored varying Aspirin concentrations (2 µm/20 µm/200 µm/2 mM/20 mM) on mouse intestinal organoids culture (Fig.S1F, G). And found that 2 mM Aspirin is the optimal concentration for aged mouse intestinal organoids forming and budding, which was consistent with reported (Dunbar et al., 2021).*

*3.* ***Dose-response effect:*** *The concentration difference reflects the distinct physiological and metabolic characteristics of Drosophila and mice. The lower concentration in Drosophila is due to its more sensitivity in Aspirin, while the higher concentration in mouse organoids accounts for their more complex metabolic environment.*

*Once again, thanks for your suggestion. We have included this section in the Discussion of revised manuscript.*

*Fig. 1C-E, 1I, and Fig S1A*

*Fig. S1F, S1G*

10. The study only used female *Drosophila*. It is important to consider whether gender differences might influence the experimental outcomes. Further investigation into the potential gender-specific effects would strengthen the study's applicability.

*Response:*

*We sincerely apologize for not clearly explain why only use female Drosophila in our initial manuscript. Bruno et al found the male Drosophila has lower ISC activity with delay or absence of pathology, and better barrier function, even at old ages (Hudry, Khadayate, & Miguel-Aliaga, 2016). But in female Drosophila, their intestinal epithelium undergoes major deterioration during aging, driven by higher ISC activity, which is predictive of death (Clark et al., 2015; Rera, Clark, & Walker, 2012). Therefore, we mainly focus on studying the aged ISCs differentiation with female Drosophila in our initial manuscript.*

*However, we also have recognized the importance of potential gender-specific effects. In the revised manuscript, we repeated all lifespan analyses with male Drosophila. The outcomes mirrored those obtained from females:* *Aspirin treatment and PEX5 overexpression extended lifespan, whereas Pex5 knockdown shortened it. These data indicate that the observed effects are sex-independent. Once again, we thank you for this constructive suggestion, and more gender related will be explored in the future.*

*Aspirin and PEX5* *gene manipulation have no gender differences on Drosophila lifespan analyses*

11. The manuscript reports that overexpression of Pex5 extends the lifespan of Drosophila. Given the relatively short lifespan of fruit flies, it would be interesting to explore whether similar genetic interventions could have comparable effects in mice. Additionally, considering Aspirin's widespread use, it would be valuable to investigate whether its administration can extend the lifespan of mice.

*Response:*

*Thank you for your feedback.*

***In terms of*** ***administration Aspirin can extend the lifespan of mice****: In our study, we have proved that Aspirin has an anti-aging effect on aged mouse intestinal organoids, as rescuing its budding defects (Fig. 1J-1M). Besides, it has been reported that administration Aspirin (p=0.01) led to a significant increase in survival of male mice (Strong et al., 2008), which is consistent with our result in Drosophila. Therefore, we can confirm that Aspirin administration can extend the lifespan of mice.*

***In terms of overexpressing Pex5 extends the lifespan of mice****: In our study we have confirm the expression of PEX5 in the aged Drosophila esg^+^ cells (Fig.S2A-S2B), aged mouse colon tissue (Fig.3O-3P) and colon organoid (Fig.3Q-3R) are all increased after Aspirin treatment. And Aspirin did not rescue the differentiation defects caused by Pex5 RNAi in Drosophila midguts (Fig.4F-4G) and colon organoid (Fig.S3A-C). Thus, these results demonstrated that Aspirin's anti-aging role is strictly PEX5-dependent. Therefore, we can speculate that overexpressing Pex5 all can also extend the lifespan of mice. Nevertheless, we recognize the importance of exploring overexpressing Pex5 in mouse models to broad its applicability and clinical relevance. Thus, we will investigate whether Pex5 overexpression would extends lifespan in mice in the future research. Thank you once again for your valuable feedback on our work.*

*Fig. 1j-1m*

*Fig.S2A-S2B*

*Fig.3O-3T*

*Fig.4F-4G*

*Fig.S3A-S3C*

12. While fatty acid oxidation predominantly occurs in mitochondria, very long-chain fatty acids (VLCFAs) are also known to be metabolized in peroxisomes. In Figure 6, fatty acid detection analyses of mouse intestinal tissues suggest the accumulation of VLCFAs in aging cells. However, the data presented do not address the subcellular localization of these fatty acids. Therefore, the statement "the peroxisomal β-oxidation of VLCFAs" lacks sufficient supporting evidence, as the intracellular compartmentalization of the fatty acids remains unclear.

*Response:*

*Fatty acids are constituent components of lipids and they are generally categorized based on their carbon chain lengths, VLCFAs are fatty acids with ≥20 carbon atoms. The location of VLCFAs can be found in cytoplasm, but the oxidation of VLCFAs occurs exclusively in peroxisomes (Di Cara et al., 2023; Wanders, Baes, Ribeiro, Ferdinandusse, & Waterham, 2023). Therefore, peroxisome is the only places for VLCFAs oxidation. While existing techniques, such as fluorescence microscopy imaging and mass spectrometry imaging (Guo et al., 2023; Hostetler, Kier, & Schroeder, 2006) permit fatty acid detection in situ, the limited abundance of VLCFAs poses challenges in achieving precise intracellular compartmentalization within peroxisomes. Therefore, the intracellular compartmentalization of the fatty acids remains challenge for us. Once again, thanks for your suggestion.*

13. This study primarily investigates the role of fatty acid metabolism in intestinal aging in mice. However, no comprehensive analysis of other lipid classes (e.g., glycerides, phospholipids) is presented. Given the narrow focus on fatty acid oxidation, the term "Lipid Homeostasis" in the title of the manuscript may not be fully appropriate, as it implies a broader scope of lipid regulation that is not addressed in the current study.

*Response:*

*Thank you for your feedback. We agree with your assessment that the original title may imply a broader scope of lipid regulation than what is addressed in the current study. In response to your suggestion, we have revised the title to accurately focus on fatty acid metabolism. Now, the revised title is: "Aging-Related Peroxisomal Dysregulation Disrupts Intestinal Stem Cell Differentiation via Modulating Very Long-Chain Fatty Acid Metabolism". Thank you for your suggestion.*

Minor Comments:

1. The labeling in Figure S1D is unclear. It is recommended to clearly indicate whether Aspirin was administered or not above the images to avoid confusion.

*Response:*

*Thank you for your suggestion. We apologize for the confusion in the previous Figure S1D. Actually, they are only representative images of flies after consuming a non-absorbed food dye. By analyzing proportion of “Smurf (+)” flies, we evaluate the intestinal barrier function with or without Aspirin feeding. As labeled the panels: Smurf positive flies are shown on the left indicating destroyed intestinal barrier function, and Smurf negative flies on the right indicating normal intestinal barrier function. Once again, we appreciate your guidance.*

*Fig. S1D*

2. In Figure 2J, it is not clear whether the structures shown are indeed colonic crypts. Additionally, the lack of statistical results weakens the interpretation of the data. Providing clear identification and statistical analysis would enhance the credibility of the findings.

*Response:*

*Thank you for your feedback.* *We appreciate your comments with transmission electron microscope (TEM) to confirm the structures of colonic crypts and include statistical results in Figure 2. Due to ISCs reside predominantly in crypts, which are located at the bases of the intestinal glands and are identifiable by their small size, undifferentiated morphology, large nuclei, and sparse cytoplasmic organelles. Therefore, we can confirm that the structures we imaged are indeed colonic crypts. In addition, to better view the structures of ISCs and peroxisome, we re-performed this experiment, and clearly labeled the ISC, peroxisome, mitochondria and so on (Fig. 2J, 2K). Moreover, we have quantified peroxisome numbers within ISCs and confirmed that aged ISCs exhibit a significant reduction in peroxisome counts. Once again, we deeply* *appreciate your guidance.*

*Fig. 2J-2K*

3. The bar chart in Figure 6E lacks significance statistics comparing the two groups. It is essential to include these statistics to validate the differences observed.
*Response:*

*Thank you for your feedback. We acknowledge the lack of significance statistics in the bar chart of Figure 6E. To address this, we have performed a standard t-test to compare the two groups as below and have include the statistical results in the revised manuscript.*

*Fig. 6E*

4. In Figure 7A, the indication of positive cells is inaccurate. Correcting this would improve the clarity and accuracy of the figure.

*Response:*

*Thank you for your feedback. We appreciate your attention to indicated the correct esg^+^Pdm1^+^ cells in Figure 7A. In the revised manuscript we have corrected it as below. Once again, thank you for your suggestion.*

*Fig. 7A*

5. In Figure 5A, the quality of the representative figure is poor, it does not correspond to the statistics, and the arrows are inaccurately indicated

*Response:*

*Thank you for your feedback. We appreciate your comments regarding the quality of the* *Figure 5A. In response, we have provided new representative images that better correspond to the statistical data. Additionally, we have corrected the labeling of pre-EC cells. Once again, thank you for your suggestion to improve our study.*

*Fig. 5A*

**Reference:**

Clark, R. I., Salazar, A., Yamada, R., Fitz-Gibbon, S., Morselli, M., Alcaraz, J., . . . Walker, D. W. (2015). Distinct Shifts in Microbiota Composition during Drosophila Aging Impair Intestinal Function and Drive Mortality. *Cell Rep, 12*(10), 1656-1667. doi:10.1016/j.celrep.2015.08.004

Di Cara, F., Rachubinski, R. A., & Simmonds, A. J. (2019). Distinct Roles for Peroxisomal Targeting Signal Receptors Pex5 and Pex7 in Drosophila. *Genetics, 211*(1), 141-149. doi:10.1534/genetics.118.301628

Di Cara, F., Savary, S., Kovacs, W. J., Kim, P., & Rachubinski, R. A. (2023). The peroxisome: an up-and-coming organelle in immunometabolism. *Trends Cell Biol, 33*(1), 70-86. doi:10.1016/j.tcb.2022.06.001

Drew, D. A., Cao, Y., & Chan, A. T. (2016). Aspirin and colorectal cancer: the promise of precision chemoprevention. *Nat Rev Cancer, 16*(3), 173-186. doi:10.1038/nrc.2016.4

Du, G., Liu, Z., Yu, Z., Zhuo, Z., Zhu, Y., Zhou, J., . . . Chen, H. (2021). Taurine represses age-associated gut hyperplasia in Drosophila via counteracting endoplasmic reticulum stress. *Aging Cell, 20*(3), e13319. doi:10.1111/acel.13319

Du, G., Qiao, Y., Zhuo, Z., Zhou, J., Li, X., Liu, Z., . . . Chen, H. (2020). Lipoic acid rejuvenates aged intestinal stem cells by preventing age-associated endosome reduction. *EMBO Rep, 21*(8), e49583. doi:10.15252/embr.201949583

Du, G., Xiong, L., Li, X., Zhuo, Z., Zhuang, X., Yu, Z., . . . Chen, H. (2020). Peroxisome Elevation Induces Stem Cell Differentiation and Intestinal Epithelial Repair. *Dev Cell, 53*(2), 169-184 e111. doi:10.1016/j.devcel.2020.03.002

Dunbar, K., Valanciute, A., Lima, A. C. S., Vinuela, P. F., Jamieson, T., Rajasekaran, V., . . . Din, F. V. N. (2021). Aspirin Rescues Wnt-Driven Stem-like Phenotype in Human Intestinal Organoids and Increases the Wnt Antagonist Dickkopf-1. *Cell Mol Gastroenterol Hepatol, 11*(2), 465-489. doi:10.1016/j.jcmgh.2020.09.010

Gould, S. J., Krisans, S., Keller, G. A., & Subramani, S. (1990). Antibodies directed against the peroxisomal targeting signal of firefly luciferase recognize multiple mammalian peroxisomal proteins. *J Cell Biol, 110*(1), 27-34. doi:10.1083/jcb.110.1.27

Guo, X., Wang, X., Tian, C., Dai, J., Zhao, Z., & Duan, Y. (2023). Development of mass spectrometry imaging techniques and its latest applications. *Talanta, 264*, 124721. doi:10.1016/j.talanta.2023.124721

Hostetler, H. A., Kier, A. B., & Schroeder, F. (2006). Very-long-chain and branched-chain fatty acyl-CoAs are high affinity ligands for the peroxisome proliferator-activated receptor alpha (PPARalpha). *Biochemistry, 45*(24), 7669-7681. doi:10.1021/bi060198l

Hudry, B., Khadayate, S., & Miguel-Aliaga, I. (2016). The sexual identity of adult intestinal stem cells controls organ size and plasticity. *Nature, 530*(7590), 344-348. doi:10.1038/nature16953

Luo, T., Zhao, L., Feng, C., Yan, J., Yuan, Y., & Chen, H. (2025). Asparagine prevents intestinal stem cell aging via the autophagy-lysosomal pathway. *Aging Cell, 24*(4), e14423. doi:10.1111/acel.14423

Noreen, F., Chaber-Ciopinska, A., Regula, J., Schar, P., & Truninger, K. (2020). Longitudinal analysis of healthy colon establishes aspirin as a suppressor of cancer-related epigenetic aging. *Clin Epigenetics, 12*(1), 164. doi:10.1186/s13148-020-00956-9

Pentinmikko, N., Iqbal, S., Mana, M., Andersson, S., Cognetta, A. B., 3rd, Suciu, R. M., . . . Katajisto, P. (2019). Notum produced by Paneth cells attenuates regeneration of aged intestinal epithelium. *Nature, 571*(7765), 398-402. doi:10.1038/s41586-019-1383-0

Rera, M., Clark, R. I., & Walker, D. W. (2012). Intestinal barrier dysfunction links metabolic and inflammatory markers of aging to death in Drosophila. *Proc Natl Acad Sci U S A, 109*(52), 21528-21533. doi:10.1073/pnas.1215849110

Strong, R., Miller, R. A., Astle, C. M., Floyd, R. A., Flurkey, K., Hensley, K. L., . . . Harrison, D. E. (2008). Nordihydroguaiaretic acid and aspirin increase lifespan of genetically heterogeneous male mice. *Aging Cell, 7*(5), 641-650. doi:10.1111/j.1474-9726.2008.00414.x

Walker, C. L., Pomatto, L. C. D., Tripathi, D. N., & Davies, K. J. A. (2018). Redox Regulation of Homeostasis and Proteostasis in Peroxisomes. *Physiol Rev, 98*(1), 89-115. doi:10.1152/physrev.00033.2016

Wanders, R. J. A., Baes, M., Ribeiro, D., Ferdinandusse, S., & Waterham, H. R. (2023). The physiological functions of human peroxisomes. *Physiol Rev, 103*(1), 957-1024. doi:10.1152/physrev.00051.2021

Yan, L., Guo, X., Zhou, J., Zhu, Y., Zhang, Z., & Chen, H. (2022). Quercetin Prevents Intestinal Stem Cell Aging via Scavenging ROS and Inhibiting Insulin Signaling in Drosophila. *Antioxidants (Basel), 12*(1). doi:10.3390/antiox12010059

Yu, Z., Zhu, Y., Chen, Y., Feng, C., Zhang, Z., Guo, X., . . . Chen, H. (2024). Nutrient-sensing alteration leads to age-associated distortion of intestinal stem cell differentiating direction. *Nat Commun, 15*(1), 9243. doi:10.1038/s41467-024-53675-7

Zhang, Q., Deng, K., Liu, M., Yang, S., Xu, W., Feng, T., . . . Jiang, H. (2023). Phase separation of BuGZ regulates gut regeneration and aging through interaction with m(6)A regulators. *Nat Commun, 14*(1), 6700. doi:10.1038/s41467-023-42474-1

Zhao, L., Luo, T., Zhang, H., Fan, X., Zhang, Q., & Chen, H. (2024). Kaempferol enhances intestinal repair and inhibits the hyperproliferation of aging intestinal stem cells in Drosophila. *Front Cell Dev Biol, 12*, 1491740. doi:10.3389/fcell.2024.1491740

Zhu, Y., Cai, Q., Zheng, X., Liu, L., Hua, Y., Du, B., . . . Ji, S. (2021). Aspirin Positively Contributes to Drosophila Intestinal Homeostasis and Delays Aging through Targeting Imd. *Aging Dis, 12*(7), 1821-1834. doi:10.14336/AD.2020.1008

Zhu, Y., Wang, F., Zhao, Y., Wang, P., & Sang, S. (2017). Gastroprotective [6]-Gingerol Aspirinate as a Novel Chemopreventive Prodrug of Aspirin for Colon Cancer. *Sci Rep, 7*, 40119. doi:10.1038/srep40119
