# Supplementary material for: Aging-related peroxisomal dysregulation disrupts intestinal stem cell differentiation through alterations of very long-chain fatty acid oxidation
Source: PLoS Biol. 2025 Dec 19;23(12):e3003552. doi: 10.1371/journal.pbio.3003552 (PMC12716710; doi:10.1371/journal.pbio.3003552)
Supplement: Attachment — Submitted filename: Response_letter_to_Reviewers_auresp_3.docx [file pbio.3003552.s025.docx]

**Reviewers' comments**
**Rev. 1:**
The authors have adequately addressed all of my concerns. The revisions have led to significant improvements in the manuscript.

***Response:***

*We appreciate your valuable input on our paper and are grateful for your recognition that we have adequately addressed the reviewers' concerns.*

**Academic Editor's comments**
The author has largely answered my concerns, but there are a few other comments, which I believe will make the manuscript more convincing.
***Response:***

*Thank you for your constructive feedback and for recognizing the improvements we have made in our manuscript. In this revised manuscript, we have addressed the issues you raised regarding replace the repeated t-tests with an ANOVA (or its non-parametric equivalent) across more than two groups. In accordance with your comment, we systematically evaluated the normality assumption using the Shapiro-Wilk test not only for multi-group comparisons but also for all two-group analyses. We trust that these adjustments will resolve your concerns.*

1.Inter-group comparisons involve >2 conditions, yet no omnibus test (e.g., one-way ANOVA or Kruskal-Wallis) was applied; instead, only pairwise t-tests are reported. This approach inflates the type-I error rate and is therefore inappropriate. Please replace the repeated t-tests with an ANOVA (or its non-parametric equivalent) followed by a valid post-hoc multiple-comparison procedure.
***Response:***

*We thank the Academic Editor for this important comment. We fully agree and apologize for the statistical oversight. In the revised manuscript, we firstly performed the Shapiro-Wilk normality test to confirm all for normal data, then using the two-step approach across more than two groups as below:*

*1. Overall testing: Used one-way ANOVA (for normal data) or Kruskal-Wallis test (for non-normal data) to first assess overall significance.*

*2. Post-hoc testing: Where the overall test was significant (P < 0.05), we performed Tukey's test (after ANOVA) or Dunn's test (after Kruskal-Wallis) for pairwise comparisons, and then reporting adjusted P values.*

*This approach properly controls the Type I error rate and strengthens our conclusions. Besides, all statistical data and approach have been added into supplementary files as (S1 Data to S7 Data). We appreciate your time and consideration.*

2.The assumption of normality must be explicitly verified or justified before any parametric test, including t-tests, is used. Otherwise, non-parametric alternatives should be adopted.

***Response:***

*We thank the Academic Editor for this attentive comment. In accordance you’re your comment, we systematically evaluated the normality assumption using the Shapiro-Wilk test not only for multi-group comparisons but also for all two-group analyses. Based on the test results for two-group analyses: (a) datasets conforming to a normal distribution (P > 0.05) were analyzed using the parametric unpaired t-test; (b) while datasets significantly deviating from normality (P ≤ 0.05) were analyzed with the non-parametric Mann-Whitney test. We are grateful for this insightful suggestion, which has enhanced the statistical rigor of our manuscript. Once again, we thank you for the valuable feedback, which has further improved our manuscript.*

3.The heatmaps lack a title for the color legend, such as 3B, 3T, and 6C, which makes it unclear what the color scale represents (e.g., expression level, fold change, or z-score). It is recommended to add an appropriate legend title to clarify the meaning of the color gradient.
***Response:***

*Thanks for your suggestion. We agree that clarifying the color legend is essential for interpreting the heatmaps. In Figures 3B, 3T, and 6C, the color legend all indicated as "Z-score" and we have added it in the revised manuscript.*

*Figures 3B, 3T, and 6C*
